# MicROS-drt: supporting real-time and scalable data distribution in distributed robotic systems

**DOI:** 10.1186/s40638-016-0038-y

**Published:** 2016-05-12

**Authors:** Bo Ding, Huaimin Wang, Zedong Fan, Pengfei Zhang, Hui Liu

**Affiliations:** College of Computer, National University of Defense Technology, Changsha, Hunan China

**Keywords:** Real-time data distribution, Robot software infrastructure, Distributed computing

## Abstract

A primary requirement in distributed robotic software systems is the dissemination of data to all interested collaborative entities in a timely and scalable manner. However, providing such a service in a highly dynamic and resource-limited robotic environment is a challenging task, and existing robot software infrastructure has limitations in this aspect. This paper presents a novel robot software infrastructure, micROS-drt, which supports real-time and scalable data distribution. The solution is based on a loosely coupled data publish-subscribe model with the ability to support various time-related constraints. And to realize this model, a mature data distribution standard, the data distribution service for real-time systems (DDS), is adopted as the foundation of the transport layer of this software infrastructure. By elaborately adapting and encapsulating the capability of the underlying DDS middleware, micROS-drt can meet the requirement of real-time and scalable data distribution in distributed robotic systems. Evaluation results in terms of scalability, latency jitter and transport priority as well as the experiment on real robots validate the effectiveness of this work.

## Introduction

**Fig. 1:**
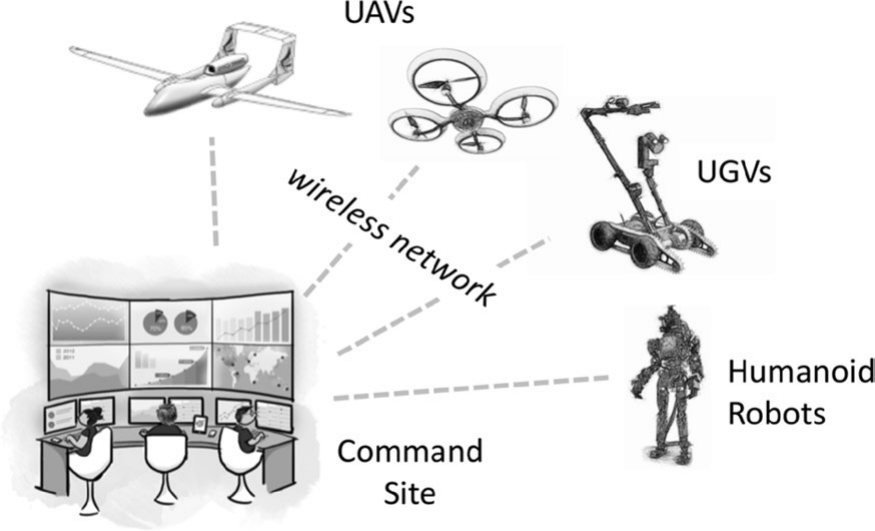
Motivated urban search and rescue scenario

Consider the following robot-assisted urban search and rescue (USAR) [1] scenario. A large-scale region should be explored after an earthquake to analyze the disaster situation and localize the victims. A team composed of several human operators, and a group of rescue robots is sent to execute this task. As shown in Fig. 1, those robots are tele-operated by the human operators through a wireless network with limited bandwidth. The human operators monitor the behavior of the robots and the video captured by them, analyze the collected data and guide the actions of the robots remotely.

This scenario involves a distributed robotic system in which various kinds of data have to be shared among participants, such as the video captured by the robots and the control commands issued by the operators. It implies some requirements to the data delivery service. Firstly, the data distribution process should be decoupled between senders and receivers because the computing environment is highly dynamic. Secondly, multiple data streams are found in this scenario, and different data streams may have different time-related constraints. For instance, the control commands to the robots issued by the human operators should be delivered in a high priority, and the video captured by the robots can be delayed or even dropped if the network capacity is not adequate. Moreover, the data distribution scalability in terms of processing and network bandwidth overhead is a crucial issue since there are a lot of participants and the network resources are limited.

The above-mentioned requirements illustrate the concern of this paper, that is, the real-time and scalable data distribution in distributed robotic systems. Here, the term “real time” means disseminating data along with specific time-related constraints [2], such as predictable latency or a certain priority in data distribution. And the term “scalable” lays emphasis on the efficiency of this process, especially when there are a lot of participants. Realizing those two goals in a distributed robotic system is usually highly dynamic and resource limited.

Data distribution is a basic research topic in distributed computing, and there has been much progress in real-time and scalable data distribution in the traditional distributed computing systems [3]. However, little prior work has been done to adapt them into robotic settings, especially to integrate them into robotic software infrastructure. As shown in the detailed presentation of related work in  “Related work” section, most of the widely accepted robot software infrastructure, such as the robot operating system (ROS) [4] and the Open Robot Control Software (OROCOS) [5], does not support real-time data distribution across computing nodes yet. This has been a serious impediment to the development and further application of distributed robotic systems.

This paper presents the design and implementation of micROS-drt, a software infrastructure that supports real-time and scalable data distribution in distributed robotic systems. A loosely coupled data publish-subscribe model for robots is proposed firstly. In this model, two kinds of message topics are defined: *general topics* without real-time assurance and *real*-*time topics* which support the fine definition of transport priority, latency budget, time-based filter and other real-time parameters. To reify this model, a mature data distribution standard, Object Management Group (OMG)’s data distribution service for real-time systems (DDS) [6], is adopted as the foundation of the transport layer of micROS-drt. We adapt and encapsulate the capability of the underlying DDS middleware to meet the requirements in robotic data distribution we mentioned earlier. Evaluation results in terms of scalability, latency jitter and transport priority on a test bed, as well as the experiment on real robots, show that micROS-drt can disseminate data scalably with real-time constraints in distributed robotic systems.

The remainder of this paper is organized as follows: “Research background” section introduces the research background. “Real‑time data distribution model for robots” section proposes a real-time data distribution model for robots.  “MicROS‑drt: architecture and implementation” section introduces the architecture of micROS-drt, as well as highlights some implementation details.  “Experiments and evaluation” section focuses on the experiments on the test bed and real robots.  “Related work” section presents related work.

## Background

This section discusses the requirements of distributed real-time computing in robotic settings firstly and introduces two software entities highly related to our work (i.e., DDS and ROS).

### Robotic distributed real-time computing

A real-time system is a system whose correctness depends not only on the logical correctness of the system but also on the time at which the results are produced [2]. Given that a robot is an autonomous agent that closely bound to the physical world, the time limit that exists in the physical space will be directly mapped to the robot software. Therefore, time-related constraints such as deadline or priority play significant roles in the logical correctness of a robot software system. The concept of robotic distributed real-time computing can be obtained by applying those real-time constraints to a complex robot made up of multiple computing nodes or a group of networked robots. The motivated scenario in “Introduction” section is a typical example of this concept.

**Fig. 2:**
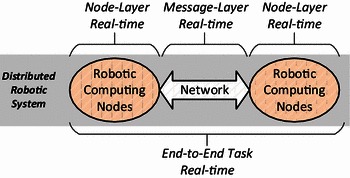
Node layer, message layer and end-to-end task real-time

The realization of the real-time assurance in a non-distributed environment mainly depends on appropriate scheduling of various local computing resources, such as the CPU or I/O devices. In contrast, its assurance in a distributed computing environment is much more complex. The dependencies among different processors and network resources should be considered, given that a task is accomplished by the collaboration of a set of nodes. In concrete, the realization of real-time properties in a distributed computing environment can be divided into three layers (Fig. 2):

*Layer 1 Node*-*level real*-*time* is concerned with scheduling the local computing resources inside a single robotic computing node.

*Layer 2 Message*-*level real*-*time* is concerned with scheduling the resources on the network which connects the robotic computing nodes together. Its goal is to ensure the message-related real-time properties such as the latency or priority of data transportation.

*Layer 3 End*-*to*-*end task real*-*time* includes but not limited to the former two layers. In addition to those two layers, the real-time constraints such as task priority and deadline should be able to be propagated among all participant entities to avoid priority inversion [2].

In this paper, we mainly focus on Layer 2, that is, the timely dissemination of data among the nodes in the robotic distributed computing.

### Data distribution service for real-time systems

DDS [6] is a message-oriented middleware standard proposed by OMG. It adopts a topic-based publish-subscribe communication model, in which the data producer (i.e., the publisher) does not send data directly to its consumers (i.e., the subscribers). Instead, the data are published into a channel with a specified “topic” name; all consumers who subscribe this topic receive the data without knowledge of who published them. The publisher and the subscriber are decoupled in terms of both time and space, which is suitable for highly dynamic environment such as robotic distributed computing systems.

In contrast to other message-oriented communication standards such as Advanced Message Queuing Protocol (AMQP) [7], DDS has the following two prominent features: (1) *Quality of Service* (*QoS*) *support*. DDS supports the fine control over various QoS parameters related to time constraints, including deadline, latency budgets, delivery order and priority and (2) *Scalability*. DDS adopts a peer-to-peer model with a fully decentralized architecture unlike many message-oriented middleware, which has a centralized broker. And the support of UDP/IP multicast also makes it scalable when the number of subscribers in a topic is more than one. Moreover, DDS is a mature and industrialized standard. Both commercial and open-source DDS middleware have widely been applied into many real production systems, such as aerospace, defense, industrial automation and cloud computing systems [3, 8].

The above-mentioned features are the reasons for our adoption of the DDS middleware as the foundation of micROS-drt transportation layer. The adoption of DDS middleware is the root of the scalability and the real-time capability of micROS-drt.

### Robot operating system

ROS [4] is an open-source robot software infrastructure being maintained by the Open Source Robotics Foundation (OSRF). It is a meta-operating system, which means that it runs on top of the existing operating systems such as Ubuntu. By adding this additional software layer, the standardized ROS programming model in which the minimal software unit is named as “package” can be supported; thus, reuse in robot software development can be promoted. Another main feature of ROS is that it can support the distributed message publish-subscribe model (without real-time assurance), which facilitates the development of loosely coupled distributed robot software.

ROS has exerted considerable influence in the robot community, and a successful ROS-based software ecosystem has been developed. Thousands of reusable software packages and numerous tools necessary for robotic research have been accumulated on top of this platform. Thus, a major design consideration of micROS-drt is to maintain compatibility with the existing ROS packages.

## Methods

### Real-time data distribution model for robots

**Fig. 3:**
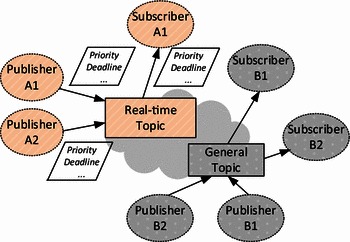
Real-time data distribution model for robots

We propose a loosely coupled, topic-based data publish-subscribe model with real-time assurance as the foundation of our work. As shown in Fig. 3, there are two kinds of topics: g*eneral topics* without real-time support and *real*-*time topics*. Unlike a general topic, various time-related QoS parameters can be specified in a real-time topic, and micROS-drt is expected to provide corresponding support while delivering data in this topic. Reviewing the motivated scenario enables the capture of the following real-time properties in robotic data distribution: (1) Different data streams may have different transport priorities or desired latency. (2) Since the network resource is limited, we should provide means to avoid network congestion, such as automatically dropping the out-of-date messages or time-based message filtering. Therefore, four kinds of time-related QoS parameters are supported in our model:

#### Transport priority/latency budget

By specifying the message transport priority or its latency budget, the transport layer of micROS-drt can schedule its network resources accordingly and decide which one should be sent firstly. Transport priority/latency budget is the most useful real-time parameter in many real-time distributed robotic systems.

#### Message auto-discarding

A message can have a lifespan (i.e., valid period). It can be automatically discarded by micROS-drt when this time period has expired and the subscriber has not received it. This feature reduces the network traffic under certain circumstances. For instance, the out-of-date video frames can be automatically dropped when a network congestion takes place in the motivated scenario.

#### Time-based message filtering

The subscriber can specify a desired time interval of the message arriving in a topic by introducing a time-based filter. The messages that arrive ahead of the time interval are dropped to avoid wasting memory and computing resources. This filtering is useful when the software needs to handle periodic messages, such as the data from a specific sensor.

#### Data transport reliability

When selecting the best-effort transport means instead of the reliable one, data are delivered without arrival checks and lost data on the network are not re-transmitted. This parameter is useful when the network resource is limited and the data reliability is not a major concern.

### MicROS-drt: architecture and implementation

This section provides an overview of micROS-drt, which includes its major design considerations and high-level architecture. This section also highlights some implementation details.

#### Design considerations

As we have stated, micROS-drt is designed to be a robot software infrastructure that supports real-time and scalable data distribution. A number of design considerations are accounted for as described below:

1. *Open source.* By adopting the open-source paradigm, we can take advantage of the community’s force to improve our work and contribute to the community effectively as well. The existing achievements in the open-source society can also be reused with appropriate copyright license.
2. *Usability.* DDS has shown its potential in real-time data distribution. However, an easy target for blame is its complex, hard-to-use APIs. A design goal of micROS-drt is to seek an appropriate trade-off between usability and flexibility. “Real-time data distribution APIs” section offers more details on this design consideration.
3. *Compatibility.* As mentioned in “Robot operating system” section, there have been thousands of reusable software packages on top of the ROS platform. Keeping compatibility with these packages is a major concern in designing and realizing micROS-drt.

#### Architecture of micROS-drt

**Fig. 4:**
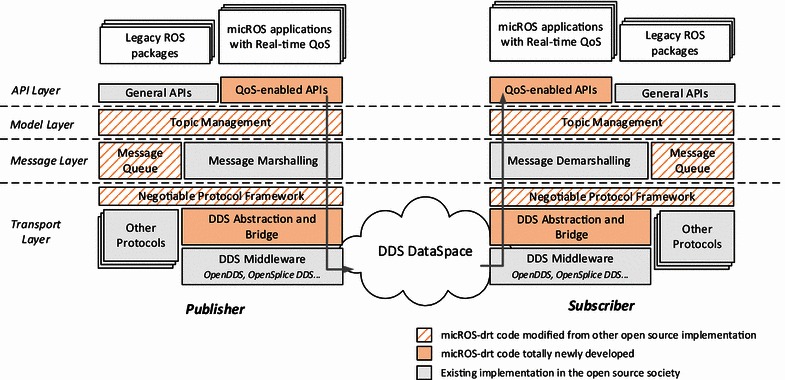
Overall architecture of micROS-drt

The architecture of micROS-drt is shown in Fig. 4. The top layer in this architecture is *the API Layer*, which provides the application programming interfaces to the robot applications. In corresponding to the two kinds of topics in the data distribution model, there are two kinds of APIs: the general data pub/sub APIs and the real-time QoS-enabled APIs. *The model layer* and *the message layer* are responsible for maintaining the data distribution model and marshaling/demarshaling the messages, respectively. The bottom layer is *the transport layer*, which consists of the negotiable transport protocol framework and a set of concrete protocols. The most important protocol is the DDS one, whose realization is based on a piece of DDS middleware.

The realization of micROS-drt is based on two open-source software entities: ROS and the DDS-compliant middleware. The topic layer and the message layer in Fig. 4 are an enhancement of the corresponding component of ROS, in which the support of the real-time topics is added. The transport layer is a mixture of the DDS Abstraction and Bridge module, the open-source DDS middleware and the existing ROS transport protocols. Theoretically, any realization of the DDS standard can be used. Currently, micROS-drt support OpenDDS (www.opendds.org) and OpenSplice DDS (www.opensplice.org).

#### Real-time data distribution APIs

The DDS standard, which has been denounced by the users, has hard-to-use APIs. It introduces more than 20 policies to define the QoS of the data distribution process. Furthermore, different policy combinations result in different effects. To avoid confusing the users of micROS-drt with those complex policies, micROS-drt chooses not to directly expose the DDS APIs directly and introduces a more practical and simplified real-time data distribution model (cf. Sect. “Real‑time data distribution model for robots”) instead. The QoS-enabled APIs of this model are an extension of the APIs in ROS, which have been widely accepted by the robotic community. The real-time parameters are classified into two kinds: The parameters should be set at the publisher side and the ones should be set at the subscriber side. The users can specify the parameters independently in the ROS-style APIs on both sides or just use their default values while advertising or subscribing a topic.

Table 1 presents an example of the real-time data distribution APIs in micROS-drt. It is the function to advertise a topic with QoS at the publisher side. Basically, it just adds some time-related parameters to the ROS’s original *advertise()* function. In the newly introduced *advertise_qos_ops* structure, all time-related QoS parameters supported by our real-time model can be selectively specified. For example, if we want to drop out-of-date video frames automatically, we can specify the *msg_valid_time* field of this structure to an appropriate value while advertising the video topic.

**Table 1:** An example of real-time API

Function name	Overloading parameters
AdevertiseWithQoS	(topic_name, queue_size, transport_priority)
	(topic_name, queue_size, latency_budget) (topic_name, queue_size, advertise_qos_ops)
	(advertise_ops, advertise_qos_ops)

#### DDS abstraction and bridge

The implementation of the DDS protocol in micROS-drt mainly consists of two modules as shown in Fig. 4: the DDS abstraction/bridge module and the underlying DDS middleware. The former can be regarded as “glue” between other parts in micROS-drt and the DDS middleware. It consists of three sub-modules: (1) *DDS capability abstraction*, which encapsulates the capability of the underlying DDS middleware as well as manages the lifetime of all DDS-related resources; (2) *Data distribution model mapping*, which maps the micROS-drt data distribution model to the DDS data distribution model, including the mapping of both topics and QoS parameters; and (3) *Message tunneling*, which encapsulates the messages that are marshaled by the message layer into a DDS message at the publisher side and extracts it from the DDS message at the subscriber side.

A challenge in the design of the DDS abstraction and bridge module is the efficiency of message handling, especially on an onboard computer of a robot which may only have limited resources. To address this issue, we strictly constrain the capability of the data distribution model as a subset of the minimum profile of the DDS standard. Thus, the underlying DDS middleware can be tailored to fit the needs of micROS-drt.

#### Keeping compatibility with ROS

An important design consideration of micROS-drt is its compatibility with thousands of existing ROS packages. To realize this goal, as shown in “Real‑time data distribution model for robots” section, two kinds of topics are strictly distinguished in our data distribution model, and the APIs of the general topic are identical with those in ROS. Moreover, the realization of existing ROS transport protocols such as TCPROS is retained in micROS-drt. The ROS negotiable protocol framework is enhanced to support the negotiation of the newly introduced DDS protocol. At runtime, TCPROS or other available protocols are selected transparently for the general topics instead of the DDS one when the remote node (with an official ROS) does not support the DDS protocol.

## Results and discussion

A set of experiments has been conducted to evaluate micROS-drt, which include both the experiments on a dedicated test bed composed of a group of servers and the experiments on a group of real distributed robots.

### Experiments on test bed

The test bed comprises of a group of servers with Intel Xeon E5-2630v3 CPU, 8 GB RAM and Ubuntu 14.04. A 1000 Mbps LAN connects the servers together. The version of OpenDDS in our experiments is 3.1.6, the version of the OpenSplice DDS middleware is 6.4, and the version of ROS is Indigo. The experiments in this subsection mainly focus on the real-time capability of micROS-drt and the scalability of our work.

#### Throughput and scalability

**Fig. 5:**
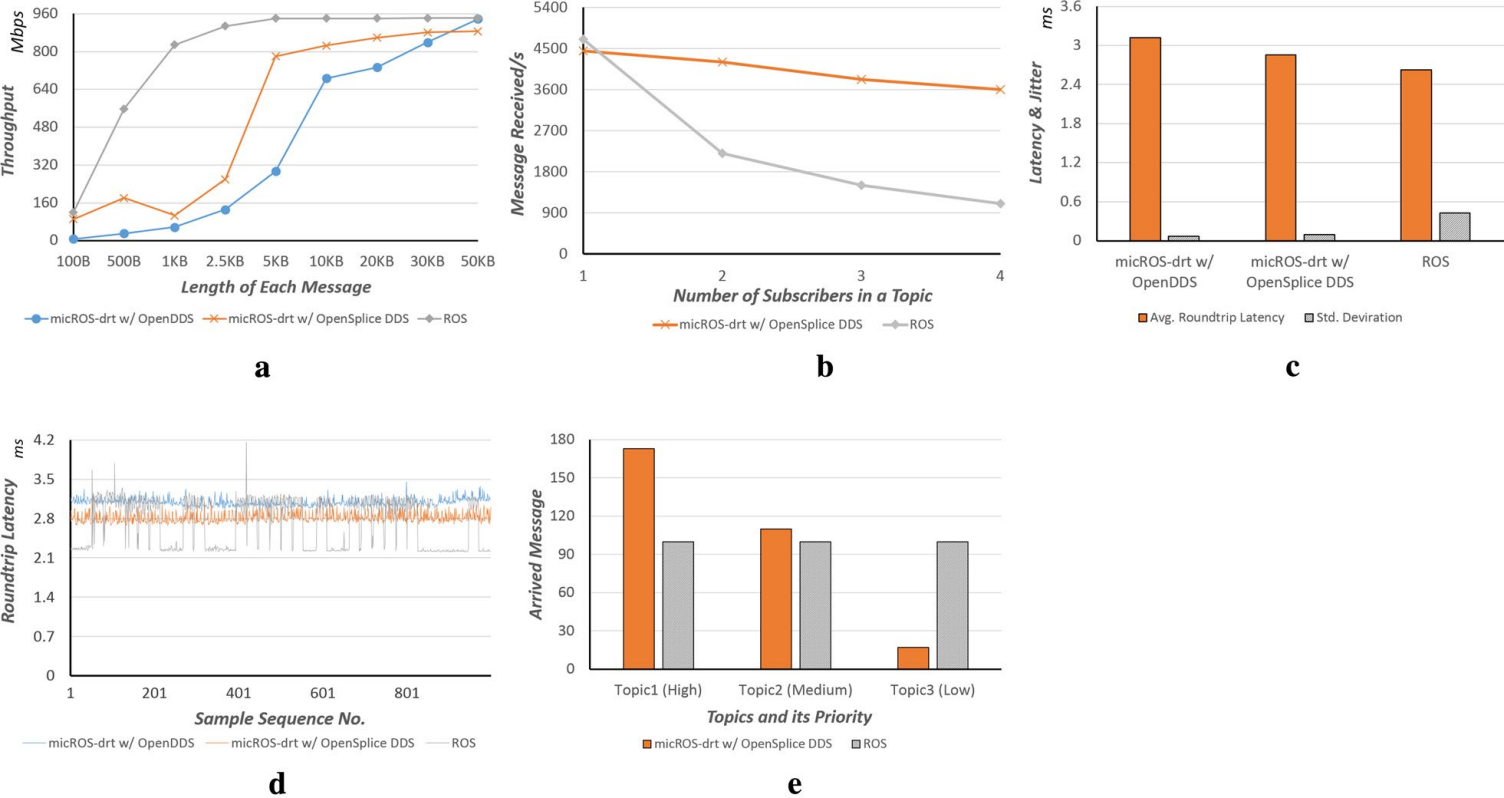
Experiment results on the test bed. **a** Throughput with different message lengths. **b** Throughput scalability. **c** Average latency and its jitter. **d** A snapshot of latency test. **e** Transport priority

In this experiment, we test the throughput of two configurations of micROS-drt (with OpenDDS and OpenSplice DDS, respectively) as well as the throughput of ROS on the test bed which has no real-time support. As shown in Fig. 5a, the “1 publisher to 1 subscriber” model is adopted (i.e., two robots are simulated), and the message length is varied from 100 B to 50 KB. The result shows that micROS-drt is slower than the official ROS to reach the limitation of the network bandwidth. It is a normal overhead incurred by both the extra real-time assurance code and the additional field in the network message.

Although micROS-drt has no advantage over ROS in terms of throughput, it has a significant advantage in terms of scalability. To evaluate it, the above throughput experiment is re-conducted with a “1 publisher to *n* subscribers” model in which *n* is a variable from 1 to 4 (i.e., the experiment simulated 5 robots in maximum). Each message is 20 KB in length. Figure 5b shows that the throughput of ROS drops drastically when the *n* increases since it does not support multicast and has to send *n* copies of the data. In contrast, the micROS-drt performance is more stable because the underlying DDS middleware supports the UDP/IP multicast. Therefore, only one copy of the data is delivered regardless of the number of subscribers.

#### Latency and jitter

Latency is an important measurement to evaluate the performance of data distribution. This experiment evaluates the message round-trip latency in micROS-drt with OpenSplice DDS, micROS-drt with OpenDDS and ROS. Each message is 100 KB in length, and 5000 messages are sent continuously. As shown in Fig. 5c, although the average latency in the three configuration/products is about the same, the latency standard deviation is totally different. The ROS latency without real-time support has a large deviation, and the latencies in the two configurations of micROS-drt are both significantly low. The snapshot of the latencies of 1000 messages in Fig. 5d also shows this trend. It indicates that the behavior of micROS-drt is more predictable, which is an important property of the real-time software.

#### Transport priority

This experiment validates a major real-time capability of micROS-drt, that is, the message transport priority management. Two servers are connected by a virtual network over the 3G cellular network. Three topics are advertised with different priorities at the publisher side, and each of them sends messages with 5 KB length continuously. Sending many messages of this kind in a short time window results in network congestion, especially with a cellular network that has limited bandwidth. The experiment is terminated when 300 messages have been received at the subscriber side, and the arrived messages in each topic are counted. Figure 5e shows that with real-time assurance support, the topic with high priority delivers 173 messages and the low priority one delivers only 17 messages successfully. As a comparison, in the same experiment on ROS which has no transport priority support, the arrived messages are distributed evenly among those three topics.

### Experiments on real robots

**Fig. 6:**
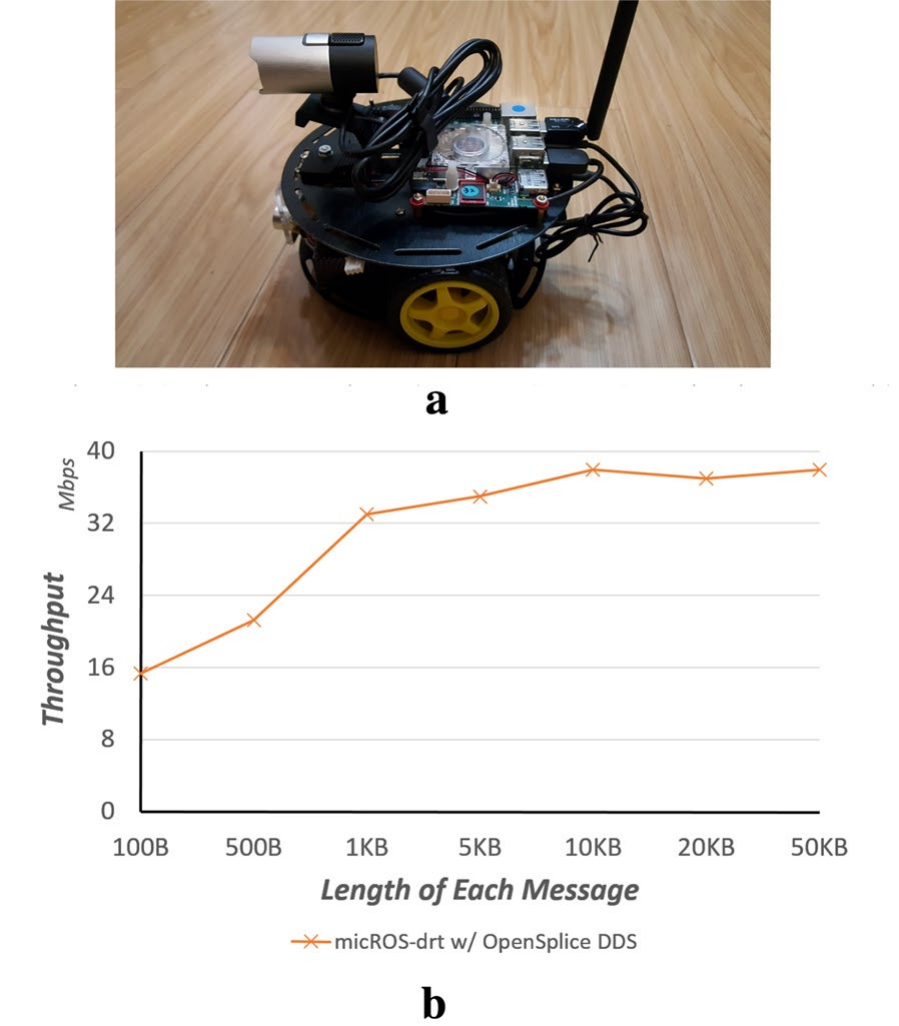
Experiment on the real robots. **a** MicROS-drt test robot (ARM CPU and Wi-Fi network). **b** Throughput on the test robots

MicROS-drt is the software infrastructure of the distributed robotic research platform in our laboratory. Figure 6a shows a compact-size three-wheel robots in this platform, which we designed mostly based on the commercial off-the-shelf hardware. The onboard computer is an ODROID XU3, a credit card-size embedded development board with a Samsung 1.8GHz ARM-based CPU. Other parts on this robot including a mbed microcontroller, two brush-less motors with encoders, an highly integrated IMU, sonar and IR sensors, and an optional general or RGB-D camera.

To maximally exploit the science research and education potential of this robot, micROS-drt is ported to it, and a software stack is constructed for this robot mainly based on existing ROS packages. With thousands of existing ROS packages, distributed or swarm robotic experiment settings (e.g., multi-robot coverage path planning [9]) can be quickly constructed. Figure 6b shows the throughput test results between two robots which are connected by an 802.11n Wi-Fi wireless network.

## Related work

In the early days, many robotic software infrastructures adopt the real-time Common Object Broker Architecture (CORBA) [10] to support robot cooperation or tele-operation, such as the work in [11] and [12]. Real-time CORBA provides a solution on real-time distributed computing. However, it is based on a tightly coupled client/server model. In contrast, our work adopts a loosely coupled publish-subscribe model, which is more suitable for dynamic scenarios. Another similar work is the Dynamic Data eXchange (DDX) project [13], which enables runtime data sharing for distributed robotic systems. DDX is realized through an efficient shared memory mechanism. However, this centralized paradigm hinders the scalability of the data distribution process. In contrast, the DDS technology we adopt has a fully decentralized architecture.

The use of publish-subscribe paradigm in robotic data distribution has recently been given increasing attention. The message transfer mechanism in ROS [4] is a typical example, which is without real-time support. Attempts have been made to integrate DDS into robot software infrastructure. In [14], DDS has been adopted to improve the performance of RoboComp, a robot software framework that originally relies on the Internet Communications Engine (ICE). A QoS-enabled middleware, Nerve, intended for networked robots and based on DDS is introduced in [15]. Since ROS has been widely accepted by the robotic community, there are also some similar attempts. In [16], the ROS–DDS proxy is introduced to support the interaction of multiple robots. It validates the feasibility to make ROS and DDS work together. However, the proxy realization is message specific. In other words, a corresponding proxy has to be developed manually for each kind of message. In contrast, our solution is general for all messages, and the DDS middleware is fully transparent to the upper layer applications. Another undertaking effort is ROS 2.0 [17], the next big step of ROS, which is expected to be released in the near future. It also adopts DDS as its underlying transport means. However, according to the information that has been disclosed such as its preview APIs [18], the real-time assurance in distributed computing environment is not its major concern.

## Conclusion

This paper presents micROS-drt, a robot software infrastructure, which supports real-time and scalable data distribution in distributed robotic computing. It is based on a loosely coupled data publish-subscribe model that supports both the topics with real-time QoS and the ones without real-time support. To reify this model, micROS-drt adopts a mature real-time data distribution standard, the OMG’s DDS, as its underlying transport means. A set of experiments on both the dedicated test bed and the real robots has validated the effectiveness of its real-time assurance capability and its scalability. In our future work, we will enhance micROS-drt to support the real-time property propagation from network resources to local computing resources.
